# Effects of Obesity Related Genetic Variations on Visceral and Subcutaneous Fat Distribution in a Chinese Population

**DOI:** 10.1038/srep20691

**Published:** 2016-02-05

**Authors:** Tao Wang, Xiaojing Ma, Danfeng Peng, Rong Zhang, Xue Sun, Miao Chen, Jing Yan, Shiyun Wang, Dandan Yan, Zhen He, Feng Jiang, Yuqian Bao, Cheng Hu, Weiping Jia

**Affiliations:** 1 Shanghai Diabetes Institute, Shanghai Key Laboratory of Diabetes Mellitus, Shanghai Clinical Center for Diabetes, Shanghai Jiao Tong University Affiliated Sixth People’s Hospital, Shanghai, 200233, China; 2 Institute for Metabolic Diseases, Shanghai Jiao Tong University Affiliated Sixth People’s Hospital South Campus, Shanghai, 201406, China

## Abstract

Genome-wide association studies (GWAS) have uncovered numerous variants associated with body mass index (BMI), waist circumference, and waist-to-hip ratio. Our study aims to investigate how these variants are linked to fat distribution. We genotyped 56 validated variants of BMI, waist circumference, and waist-to-hip ratio in 2958 subjects from Chinese community-based populations and performed linear regression analyses to determine the association with visceral fat area (VFA) and subcutaneous fat area (SFA) imaged by magnetic resonance imaging (MRI). We found rs671 in *ALDH2* exhibited the significant associations with VFA and the VFA-SFA ratio in all subjects (*P* = 9.64 × 10^−5^ and 6.54 × 10^−4^). rs17782313 near *MC4R* for VFA and rs4846567 near *LYPLAL1* for SFA were found in females only (*P* = 2.93 × 10^−4^ and 0.0015), whereas rs671 in *ALDH2* for VFA and the VFA-SFA ratio was restricted to males (*P* = 1.75 × 10^−8^ and 4.43 × 10^−8^). Given the robust association of rs671 with alcohol consumption, we next demonstrated the primary effects of rs671 on VFA and the VFA-SFA ratio were restricted to drinkers (*P* = 1.45 × 10^−4^ and 4.65 × 10^−3^). Our data implied that variants of *MC4R* and *LYPLAL1* modulated body fat distribution with sexual dimorphism and that alcohol consumption may mediate the impact of the *ALDH2* locus on visceral fat in a Chinese population.

Obesity has become a major health concern in both developed and newly emerging economies^1^. The obesity epidemic is paralleled by an increased incidence of type 2 diabetes mellitus, metabolic syndrome, and cardiovascular diseases. There is abundant evidence that central obesity, particularly intra-abdominal fat accumulation, is more responsible for morbidity and mortality in obese patients with type 2 diabetes mellitus than overall adiposity^2^,^3^.

Obesity is determined by both genetic and environmental factors. Although the “obesogenic environment” fuels the worldwide obesity epidemic, the notion that genetic variants could predispose individuals to common, polygenetic obesity seems to be an increasingly evident and persuasive argument. Certain SNPs that influence overall obesity (measured by BMI) and central adiposity (measured by the waist circumference or waist-to-hip ratio) have been identified in GWAS among European^4^,^5^,^6^,^7^,^8^,^9^,^10^,^11^,^12^,^13^,^14^,^15^ and other populations^16^,^17^,^18^,^19^,^20^,^21^,^22^,^23^,^24^. BMI, waist circumference and waist-to-hip ratio are regarded as commonly used but less precise measurements among a diverse group of obesity indices. The attempts to identify BMI loci have pointed toward the role of neuronal regulation of overall obesity^8^,^9^,^25^. Central obesity differs greatly from overall obesity in its pathogenesis, as well as in other areas; therefore, identifying the central obesity loci may aid in elucidating the signals shared with overall obesity or specific to central obesity. Fat distribution imaged by magnetic resonance imaging (MRI) is superior to waist circumference and waist-to-hip ratio in terms of distinguishing between visceral fat and subcutaneous fat. Additionally, there are few association studies of the genetic architecture of fat distribution, and the pathways determining how these variants influence the distribution of visceral and subcutaneous fat still remain unknown.

Different ethnicities have different genetic backgrounds. An indisputable fact is that large-scale obesity GWAS that include Asian and African populations are more likely to provide insight into different genetic architectures and provide evidence for fine mapping of causal genes^16^,^20^,^26^. Thus, our aim was to replicate the impact of those validated loci on BMI, waist circumference and waist-to-hip ratio in Chinese populations, which were obtained from GWAS studies of European and non-European populations. More importantly, we tested the hypothesis that precise visceral and subcutaneous fat distribution indices could provide important information beyond BMI, waist circumference, and waist-to-hip ratio with respect to identify novel variants.

## Results

### Validation of the impact of variants on BMI, waist circumference, and waist-to-hip ratio

The subject characteristics are shown in Table 1. The associations of 19 loci among 56 validated SNPs of BMI, waist circumference, and waist-to-hip ratio were well replicated in our Chinese populations (Table 2). In general, most of 19 loci showed directionally consistent effect as previous studies except for the SNPs in *ITIH4-AS1*, *MTIF3* and *ZNRF3*. The SNP rs574367 in *SEC16B* showed the most significant association with BMI in nine loci for the lowest *P* (*P* = 1.33 × 10^−6^), and the result remained significant after multiple testing correction (empirical *P* = 0.0004). The most significant association with waist circumference was observed with rs671 in *ALDH2*, with or without BMI adjustments (*P* = 1.96 × 10^−6^ and 4.05 × 10^−7^, respectively, both empirical *P* = 1 × 10^−4^). Similarly, the analysis of the waist-to-hip ratio yielded nine SNPs with nominal associations; rs17782313 near *MC4R* was the maximum signal and the risk allele carriers showed a tendency toward elevating the waist-to-hip ratio after multiple testing correction (*P* = 0.0012; empirical *P* = 0.0641).

### The association of variants with visceral and subcutaneous fat distribution

We also tested the genetic components of direct fat distribution imaged by MRI, namely, VFA, SFA, and the VFA-SFA ratio. The primary findings are presented in Table 3. Model 1 included the variables of sex and age for adjustment. Within five loci (*SEC16B*, *ETV5*, *FTO*, *ALDH2*, and *MC4R*) nominally associated with VFA, irrespective of BMI, the top locus was rs671 in *ALDH2* (*P* = 1.94 × 10^−6^). Similarly, rs574367 in *SEC16B* was the top of ten loci (including *SEC16B*, *LYPLAL1*, *TMEM18*, *RBJ*, *GRB14-COBLL1*, *NUDT3*, *ALDH2*, *MC4R*, *KCTD15*, and *ZNRF3*) for SFA (*P* = 0.0017). Two SNPs in or near *LYPLAL1* and *ALDH2* were associated with the VFA-SFA ratio, the metric describing the propensity to deposit visceral fat compared with subcutaneous fat (*P* = 0.0325 and 0.0001, respectively).

As BMI represents both fat and lean mass and correlates with regional fat depots, model 2 additionally adjusted for BMI. Although the majority of VFA signals were completely attenuated, rs671 in *ALDH2* remained unchanged (*P* = 9.64 × 10^−5^). Similarly, the SNPs in or near *RBJ* and *NUDT3* for SFA and SNPs near *LYPLAL1* and *ALDH2* for the VFA-SFA ratio also showed nominal association after adjusting for BMI (*P* = 0.01 and 0.0318 for SFA; *P* = 0.0091 and 0.0007 for VFA-SFA ratio, respectively). We also noted that SNPs in or near *POC5* and *CDKAL1* showed nominal association only with VFA after adjusting for BMI (*P* = 0.024 and 0.0318, respectively). Similarly, the SNP in or near *ITIH4-AS1* showed nominal association only with SFA after adjusting for BMI (*P* = 0.0225). Apart from the locus rs671 in *ALDH2*, none of the other loci survived the multiple comparisons (e.g., rs671 empirical *P* = 0.0043 for VFA, empirical *P* = 0.0345 for the VFA-SFA ratio).

### Gender differences in variants influence on fat distribution of visceral and subcutaneous fat

Taking into account the heterogeneity of fat distribution in both genders, we performed the male and female analyses separately, which yielded 27 SNPs associated with at least one of three traits in one gender. The association of rs671 in *ALDH2* with fat distribution traits was restricted to males (*P* = 1.75 × 10^−8^ for VFA, *P* = 4.43 × 10^−8^ for the VFA-SFA ratio, Table 4), whereas rs17782313 near *MC4R* for VFA and rs4846567 near *LYPLAL1* for SFA were only observed in females (*P* = 2.93 × 10^−4^ and 0.0015, respectively, Supplemental Table 2). All associations described above remained significant or exhibited a tendency after correction for multiple testing (empirical *P* range 1 × 10^−4^ to 0.0778). Moreover, other loci, including *CPEB*, *NRXN3*, *PPARG*, and *SPRY2,* also displayed the marked sexual dimorphism. To reduce the basis of the power loss in the subgroup analysis, we performed further joint interaction analyses of the entire group. The results indicated that the gender interaction of *ALDH2* for the VFA and VFA-SFA ratio, *MC4R* for VFA, and *LYPLAL1* for SFA remained significant (*P* for interaction range from 9.88 × 10^−8^ to 0.0398).

### Alcohol consumption mediated the effect of the *ALDH2* locus on visceral fat accumulation

**Figure 1:**
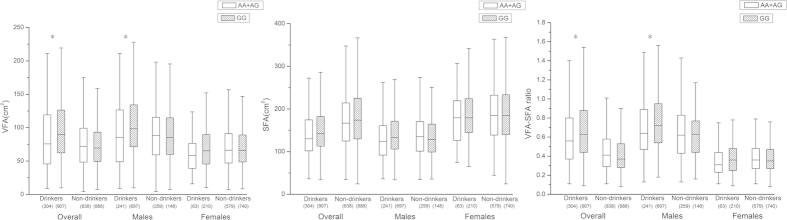
Alcohol consumption mediated the effect of the *ALDH2* locus on visceral fat accumulation. Box plots showing (**A**) The association of rs671 in *ALDH2* with VFA (**B**) The association of rs671 in *ALDH2* with SFA (**C**) The association of rs671 in *ALDH2* with the VFA-SFA ratio in a subgroup analysis stratified by alcohol consumption in overall subjects, males, and females (*P = 1.45 × 10^−4^ for VFA and 4.65 × 10^−3^ for the VFA-SFA ratio in overall drinkers, and *P = 4.22 × 10^−5^ for VFA and 0.0031 for the VFA-SFA ratio in male drinkers). The carriers of A allele (i.e. AA and AG) were merged into one group (AA + AG) because of limited number of AA individuals. VFA, SFA, and the VFA-SFA ratio among AA + AG (white) and GG (dense) genotypes of rs671 in *ALDH2* are shown as the median, quartile, minimum and maximum. The count of AA + AG (white) and GG (dense) genotypes are marked in the parentheses. *P* values were determined by linear regression under additive model adjusting for BMI additionally.

As rs671 in *ALDH2* previously demonstrated a robust association with alcohol consumption, we also confirmed the finding in our study (odds ratio 0.27, 95% confidence interval [CI] 0.09–0.23, *P* = 6.16 × 10^−46^ per copy of A allele) and then performed further analysis to evaluate the underlying effect of alcohol consumption on the association between *ALDH2* and visceral fat accumulation. While adjusting for alcohol consumption, the associations of *ALDH2* with VFA and the VFA-SFA ratio were substantially attenuated in the overall group (*P* = 0.0043 and 0.0149, respectively), as well as in males (*P* = 5.72 × 10^−5^ and 7.22 × 10^−4^). Next, we performed a subgroup analysis stratified by alcohol consumption. Data from 1211 drinkers (938 males and 273 females) and 1726 non-drinkers (407 males and 1319 females) were available, and the results are depicted in Fig. 1. Note that nominal associations between the *ALDH2* variant and visceral fat accumulation were restricted to drinkers overall (*P* = 1.45 × 10^−4^ for VFA, *P* = 4.65 × 10^−3^ for the VFA-SFA ratio) and to male drinkers specifically (*P* = 4.22 × 10^−5^ for VFA, *P* = 0.0031 for the VFA-SFA ratio). The interaction analysis of SNP × drinking revealed significant in overall individuals for VFA (*P* for interaction = 0.0055). Additionally, we also performed SNP × environment (gender × drinking) interaction analyses for rs671 in *ALDH2* and found that the SNP × environment interaction of *ALDH2* for the VFA and VFA-SFA ratio remained significant (*P* for interaction = 0.0007 and 0.0058, respectively).

In order to strengthen our finding, we performed subgroup analysis which divided subjects into three groups (i.e. non-drinkers, chance drinkers and regular drinkers). We found that the nominal associations between the *ALDH2* variant and VFA-SFA ratio were restricted to overall regular drinkers and to male regular drinkers specifically (*P* = 0.0453 and 0.0429, respectively) and that a tendency toward elevating VFA were restricted to overall regular drinkers and to male regular drinkers specifically (*P* = 0.0503 and 0.0634, respectively), but did not observe associations in chance drinkers.

## Discussion

We replicated 19 of 56 loci, such as *FTO*, *MC4R* and *KCTD15,* were nominally associated with BMI, waist circumference, and waist-to-hip ratio, but SNPs in *MC4R*, *ALDH2* and *SEC16B* were showed significant association after multiple testing correction. More importantly, in search for fat distribution variants in a Chinese population, our study revealed 15 of 56 loci nominally associated with at least one trait within three fat distribution indices, and the SNPs in or near *MC4R*, *LYPLAL1*, and *ALDH2* were significantly associated with fat distribution after multiple testing correction.

To our knowledge, this report is the first to focus on fat distribution variants in a Chinese population. Previous efforts have focused on this issue in European and other Asian populations. The results indicated that several loci, such as *LYALAL1*, *FTO*, *THNSL2*, *GCKR*, *TRIB2*, and *IRS1*, substantially impacted fat distribution indices^12^,^27^,^28^. The reported signals for fat distribution of visceral fat and subcutaneous fat from previous GWAS by the GIANT consortium such as *LYPLAL*, *TMEM18*, *GRB14-COBLL1* and *ETV5* were directionally consistent with our results^12^. Besides, their finding highlighted the associations of rs11118316 in *LYPLAL1* with the ratio of visceral fat area to subcutaneous fat area and rs1558902 in *FTO* with subcutaneous fat area. The former locus failed to be analysed for departure from Hardy-Weinberg equilibrium and the proxy of latter locus was not replicated in our study as well as in that GWAS. With the current sample size, the statistical power was 45%–95% to detect the effect size ranging 0.2 kg/m^2^ to 0.4 kg/m^2^ for BMI, more than 71% to detect the effect size ranging 0.8 cm to 1.1 cm for waist circumference, and 33%–86% to dectect the effect size ranging 0.003 to 0.006 for waist-to-hip ratio (minor allele frequency = 0.2, two-sided type one error rate = 0.05) in our study. One of the probability for negative association is thus the differences in genetic architecture among varied populations. Some variants with the modest effect size or low minor allele frequency need to be replicated in large-scale meta-analyses of GWAS across varied populations.

rs4846567 at *LYPLAL1* has been previously reported by the GIANT consortium to be associated with the waist-to-hip ratio in subjects based on GWAS, but the association was restricted to females (*P* = 2.6 × 10^−8^)^7^. Our study did not replicate this finding in the entire group or in females, but there was an association with SFA and the VFA-SFA ratio, which are consistent with the other findings in European^12^ and Japanese populations^29^. The former study also revealed the association of another independent SNP, rs11118316 near *LYPLAL1* (r^2^ = 0, D’ = 0.004 in HCB; r^2^ = 0.285, D’ = 0.935 in CEU with rs4846567) with the VFA-SFA ratio in both males and females. This SNP was not analysed in our study, but we speculated that there were heterogeneous sex-related signals associated with the VFA-SFA ratio. *LYPLAL1* encodes lysophospholipase-like protein 1, which plays a role in the consecutive steps of triglyceride degradation. This region showed an association with fasting serum triglycerides^30^, insulin resistance^31^, and non-alcoholic fatty liver disease^32^, suggesting some involvement in hepatic lipid metabolism and insulin responsiveness. The molecular mechanism responsible for the link between *LYPLAL1* and the pathogenesis of fat distribution according to gender remains to be elucidated in functional studies.

The locus rs671 in *ALDH2* was previously reported to be associated with BMI in East Asians^21^. Our novel findings were for visceral fat accumulation in overall subjects and restricted to males. However, we did note that the male to female ratio was not balanced between drinkers and non-drinkers, and the analysis of the associations of *ALDH2* with VFA and SFA revealed a borderline sex-related significance among overall drinkers (*P* for interaction = 0.0473 and 0.0406, respectively). We cannot exclude the possibility that alcohol consumption does not affect visceral fat accumulation in a sex-dependent manner. *ALDH2* encodes aldehyde dedehydrogenase-2, a mitochondrial enzyme that metabolises acetaldehyde to acetic acid and ultimately removes it^33^. Many analyses of GWAS have demonstrated the robust association of rs671 in *ALDH2* with alcohol consumption in Asian populations; however, this SNP does not appear to be polymorphic in Europeans^34^,^35^,^36^. The A allele of rs671, designated as the *ALDH*2*2 allele, encodes in an inactive form, resulting in a nearly complete loss of catalytic activity, which causes acetaldehyde-mediated “flushing syndrome” and thus acts as a preventer from alcohol consumption^33^. Therefore, we considered that rs671 in *ALDH2* may influence the visceral fat accumulation by affecting alcohol consumption, with A allele carriers having lower visceral fat depots due to lower alcohol consumption. If this hypothesis is true, reducing the alcohol consumption in individuals with a high risk of visceral fat accumulation could be more productive for obesity prevention.

There are significant differences in the pathologies and physiologies between visceral fat and subcutaneous fat accumulation. The properties of decreased insulin sensitivity, lower angiogenic potential, increased lipolytic activity, the different cellular composition, and the expression of genes regulating adipocyte function were demonstrated in visceral fat compared with subcutaneous fat^37^. Strikingly, adipose tissue deposits and function differ by sex. Males tend to accrue more visceral fat, whereas females are more likely to store subcutaneous fat before menopause and have visceral deposits after menopause^38^. It is well recognized that sex hormones contribute to this regulation^39^,^40^. Given this fact, our study uncovered several loci that are linked to fat distribution with sex dimorphism. Due to the respectively small sample size or differences in genetic architectures between European and Asian populations, our findings were not comparable with evidence that the GWAS from GIANT consortium found several loci for waist circumference and waist to hip ratio with significant sex-difference and more prominent effects in females^23^. Whether and how these loci influence fat distribution in a sex-specific manner warrant future molecular and biology investigations.

This study has several limitations. First, we did not perform further analyses after adjusting for lifestyle (e.g., alcohol consumption and smoking), except for the analysis of the *ALDH2* locus, which demonstrated a robust association with alcohol consumption. It is unknown whether there is an interaction between lifestyle and other variants on fat distribution. Second, we tested the one SNP of each locus obtained from the top signals of GWAS in varied populations, which may lead to negative findings for the lack of good coverage of the regions in Chinese. Moreover, despite the multiple comparisons performed in the study, the possibility of a spurious association still cannot be excluded.

We replicated the impacts of the loci associated with BMI, waist circumference, and waist-to-hip ratio on fat distribution in a Chinese population and demonstrated that *MC4R*, *LYPLAL1*, and *ALDH2* may modulate visceral and subcutaneous fat distribution. Our findings highlight the importance of considering direct and precise fat distribution traits in obesity-related loci investigations.

## Materials and Methods

### Subjects

From 2009–2012, we recruited up to 2958 subjects from a community-based population with Chinese Han ancestry and excluded the subjects with cancer, severe disability, or severe psychiatric disturbances. The remaining subjects provided informed consent and completed a questionnaire on their medical histories; they also underwent anthropometric measurements and laboratory examinations. The study complied with the Declaration of Helsinki and was approved by the Institutional Review Board of Shanghai Jiao Tong University Affiliated Sixth People’s Hospital.

### Phenotypes and assessment of alcohol consumption

BMI was calculated as weight (kilograms) divided by height^2^ (meters). Waist circumference was measured at the level of the umbilicus, and hip circumference was measured around the buttocks. The waist-to-hip ratio was calculated as the ratio between the waist and hip circumferences in centimetres. Each subject underwent abdominal MRI (Archive, Philips Medical System, Amsterdam, Netherlands) at the level of the umbilicus between L4 and L5 in the supine position for quantification of body fat distribution. Two trained observers used SLICE-O-MATIC image analysis software (version 4.2; Tom Vision Inc., Montreal, QC, Canada) to generate graphical displays of the imaging data and to calculate the visceral fat area (cm^2^) and subcutaneous fat area (cm^2^). If the results differed by more than 10%, a third observer who was blinded to the results reanalysed the images. As for alcohol consumption, briefly, each subject was asked whether they had ever consumed alcohol in their lifetime (chance drunk less than three times in every week and regularly drunk equal or more than three times in every week) and individuals who gave a positive answer were defined as drinkers, whereas those who gave a negative answer were non-drinkers.

### Genotyping and quality control analysis

Genomic DNA was extracted from blood samples collected from each subject. A total of 57 SNPs associated with BMI, waist circumference, and waist-to-hip ratio from previous literature (as shown in Supplementary Table 1 and Supplementary Figure 1) were selected to be genotyped using the MassARRAY Compact Analyzer (Sequenom, San Diego, CA, USA). None of the 57 SNPs failed quality control analyses, with call rates >95% and concordant rates >99%. Fifty-three subjects were excluded due to sample call rate <90%. The Hardy-Weinberg equilibrium test was performed prior to the analysis. Among the 57 SNPs, 56 SNPs were in accordance with Hardy-Weinberg equilibrium (*P* > 0.05), except for rs11118316.

### Statistical analysis

Haploview (version4.2; www.broad.mit.edu/mpg/haploview/) was used to determine the pairwise linkage disequilibrium. Using PLINK (http://pngu.mgh.harvard.edu/~purcell/plink/)^41^, logistic regression analysis was used to examine the associations between SNPs and dichotomous variables, and linear regression analysis was used to test for the effects of SNPs on quantitative traits under the additive genetic model. All analyses were adjusted for covariates, such as age, sex, and other variables, if appropriate. Waist circumference, waist-to-hip ratio, VFA, SFA, and VFA-SFA ratio were log10-transformed. Since no accurate data on type and amount of alcohol consumption, alcohol consumption was converted into a dichotomous variable that includes drinkers and non-drinkers. Multiple testing based on 10000 permutations was performed with PLINK. The statistical analyses were performed using SAS software (version 8.0; SAS Institute, Cary, NC, USA), unless otherwise specified. A two-tailed *P* value of <0.05 was considered to be significant. The power calculations were performed using Quanto software (http://biostats.usc.edu/Quanto.html, version 1.2.4, May 2009).

## Additional Information

**How to cite this article**: Wang, T. *et al.* Effects of Obesity Related Genetic Variations on Visceral and Subcutaneous Fat Distribution in a Chinese Population. *Sci. Rep.* **6**, 20691; doi: 10.1038/srep20691 (2016).

## Supplementary Material

Supplementary Information

## Figures and Tables

**Table 1:** Subject characteristics.

	Overall	Males	Females	*P* value
N(%)	2958	1352(45.71)	1606(54.29)	—
Age (years)	52.05 ± 6.93	52.11 ± 6.95	52.01 ± 6.92	0.7666
BMI (kg/m^2^)	24.44 ± 3.36	24.90 ± 3.21	24.05 ± 3.43	﹤0.001
Waist circumference (cm)	86(80,93)	88.5(83,95)	83.5(77.5,90.2)	﹤0.001
Waist-to-hip ratio	0.913(0.8728,0.955)	0.9295(0.8966,0.9645)	0.8944(0.8563,0.9406)	﹤0.001
VFA (cm^2^)	75.75(51.65,108.96)	92.48(63.60,127.88)	65.64(47.18,89.45)	﹤0.001
SFA (cm^2^)	157.27(117.99, 205.21)	130.8(102.3,169.25)	184.05(140.1,230.35)	﹤0.001
VFA-SFA ratio	0.47(0.32,0.69)	0.67(0.5,0.9)	0.35(0.26,0.47)	﹤0.001

Data are shown as the mean ± SD, median (interquartile range), or N(%).

**Table 2:** The impact of variants on BMI, waist circumference, and the waist-to-hip ratio.

SNP	Gene	Alleles	MAF	Traits	Model 1		Model 2	
					BETA ± SE	*P*	BETA ± SE	*P*
rs984222	*TBX15-WARS2*	C/G	0.41	BMI	−0.0378 ± 0.0879	0.6672		
				WC	−0.0021 ± 0.0012	0.0916	−0.0017 ± 0.0007	**0.0211**
				WHR	−0.0018 ± 0.0007	**0.0151**	−0.0016 ± 0.0007	**0.0124**
rs574367	*SEC16B*	T/G	0.2	BMI	0.5353 ± 0.1105	**1.33 × 10**^**−6**^^a^		
				WC	0.0056 ± 0.0016	**3.38 × 10**^**−4**^^b^	−0.0005 ± 0.0009	0.6158
				WHR	0.0011 ± 0.0009	0.2196	−0.0007 ± 0.0008	0.373
rs4846567	*LYPLAL1*	T/G	0.3	BMI	0.192 ± 0.0939	**0.0409**		
				WC	0.0021 ± 0.0013	0.1218	0.00002 ± 0.0008	0.9806
				WHR	−0.0007 ± 0.0008	0.3893	−0.0013 ± 0.0007	0.0681
rs6548238	*TMEM18*	T/C	0.09	BMI	−0.4554 ± 0.1546	**0.0033**		
				WC	−0.0059 ± 0.0022	**0.0066**	−0.0009 ± 0.0013	0.4991
				WHR	−0.0022 ± 0.0013	0.0831	−0.0006 ± 0.0012	0.5786
rs1057001	*TRIB2*	T/A	0.15	BMI	0.0864 ± 0.1243	0.4873		
				WC	0.0036 ± 0.0017	**0.0413**	0.0025 ± 0.0011	**0.0169**
				WHR	0.0020 ± 0.0010	0.0512	0.0016 ± 0.0009	0.0779
rs887912	*FANCL*	A/G	0.002	BMI	0.8386 ± 0.9268	0.3656		
				WC	0.0197 ± 0.0130	0.131	0.0102 ± 0.0078	0.1953
				WHR	0.0166 ± 0.0076	**0.0281**	0.0137 ± 0.0069	**0.0462**
rs2535633	*ITIH4-AS1*	C/G	0.41	BMI	0.2331 ± 0.0889	**0.0088**		
				WC	0.0035 ± 0.0013	**0.0054**	0.0008 ± 0.0008	0.2694
				WHR	0.0006 ± 0.0007	0.4327	−0.0002 ± 0.0007	0.7294
rs6931262	*RREB1*	T/C	0.2	BMI	−0.0923 ± 0.1097	0.3998		
				WC	0.0007 ± 0.0015	0.6553	0.0018 ± 0.0009	0.0545
				WHR	0.0020 ± 0.0009	**0.0294**	0.0023 ± 0.0008	**0.0042**
rs6905288	*VEGFA*	G/A	0.26	BMI	0.2671 ± 0.1009	**0.0081**		
				WC	0.0030 ± 0.0014	**0.038**	0.0001 ± 0.0009	0.9024
				WHR	0.0002 ± 0.0008	0.7774	−0.0007 ± 0.0008	0.3321
rs987237	*TFAP2B*	G/A	0.17	BMI	0.0810 ± 0.1148	0.4807		
				WC	0.0027 ± 0.0016	0.0943	0.0020 ± 0.0010	**0.0387**
				WHR	0.0010 ± 0.0009	0.2777	0.0008 ± 0.0009	0.345
rs10968576	*LRRN6C-LINGO2*	G/A	0.21	BMI	0.0504 ± 0.1082	0.6416		
				WC	−0.0009 ± 0.0015	0.5359	−0.0016 ± 0.0009	0.0901
				WHR	−0.0021 ± 0.0009	**0.016**	−0.0023 ± 0.0008	**0.004**
rs4074134	*BDNF*	A/G	0.44	BMI	−0.2247 ± 0.0884	**0.0111**		
				WC	−0.0030 ± 0.0012	**0.0156**	−0.0006 ± 0.0008	0.4287
				WHR	−0.0011 ± 0.0007	0.1369	−0.0004 ± 0.0007	0.5953
rs671	*ALDH2*	A/G	0.22	BMI	−0.2932 ± 0.1052	**0.0053**		
				WC	−0.0075 ± 0.0015	**4.05 × 10**^**−7**^^c^	−0.0042 ± 0.0009	**1.96 × 10**^**−6**^^c^
				WHR	−0.0020 ± 0.0009	**0.022**	−0.0010 ± 0.0008	0.2143
rs4771122	*MTIF3*	G/A	0.18	BMI	−0.0293 ± 0.1140	0.7969		
				WC	−0.0018 ± 0.0016	0.2532	−0.0015 ± 0.0010	0.1289
				WHR	−0.0022 ± 0.0009	**0.0173**	−0.0021 ± 0.0008	**0.0147**
rs9939609	*FTO*	A/T	0.12	BMI	0.3047 ± 0.1361	**0.0253**		
				WC	0.0037 ± 0.0019	0.0563	0.0004 ± 0.0012	0.6978
				WHR	0.0018 ± 0.0011	0.1168	0.0008 ± 0.0010	0.4545
rs17782313	*MC4R*	C/T	0.22	BMI	0.3600 ± 0.1051	**0.0006**^d^		
				WC	0.0073 ± 0.0015	**9.73 × 10**^**−7**^^c^	0.0032 ± 0.0009	**3.65 × 10**^**−4**^^e^
				WHR	0.0028 ± 0.0009	**0.0012**	0.0016 ± 0.0008	**0.0471**
rs29941	*KCTD15*	C/T	0.24	BMI	0.2437 ± 0.1017	**0.0166**		
				WC	0.0030 ± 0.0014	**0.0344**	0.0004 ± 0.0009	0.6586
				WHR	0.0010 ± 0.0008	0.2458	0.0001 ± 0.0008	0.8564
rs3810291	*TMEM160*	A/G	0.29	BMI	0.1594 ± 0.0984	0.1053		
				WC	0.0025 ± 0.0014	0.0662	0.0008 ± 0.0008	0.3265
				WHR	0.0020 ± 0.0008	**0.014**	0.0015 ± 0.0007	**0.0465**
rs4823006	*ZNRF3*	A/G	0.46	BMI	−0.1732 ± 0.0884	0.0502		
				WC	−0.0020 ± 0.0012	0.1013	−0.0002 ± 0.0008	0.7473
				WHR	−0.0016 ± 0.0007	**0.0268**	−0.0011 ± 0.0007	0.1052

SNP, single nucleotide polymorphism; Alleles, minor/major alleles; MAF minor allele frequency; SE, standard error; WC, waist circumference; WHR, waist-to-hip ratio.

Only SNPs that showed nominal significant associations with traits are shown in Table 2.

*P* values < 0.05 are shown in bold.

Traits were adjusted for age and sex in the additive genetic model 1 and adjusted for age, sex, and BMI in model 2.

^a^Empirical *P* = 0.0004.

^b^Empirical *P* = 0.0187.

^c^Empirical *P* = 1 × 10-4.

^d^Empirical *P* = 0.0326.

^e^Empirical *P* = 0.0209; Empirical *P* values were based on 10000 permutations within each trait.

**Table 3:** Influence of variants on fat distribution (i.e., visceral fat and subcutaneous fat).

SNP	Gene	Alleles	MAF	Traits	Model 1		Model 2	
					BETA ± SE	*P*	BETA ± SE	*P*
rs574367	*SEC16B*	T/G	0.2	VFA	0.0221 ± 0.0080	**0.0055**	−0.0031 ± 0.0061	0.6115
				SFA	0.0175 ± 0.0056	**0.0017**	−0.0018 ± 0.0039	0.6429
				VFA/SFA	0.0047 ± 0.0063	0.4591	−0.0013 ± 0.0062	0.8343
rs4846567	*LYPLAL1*	T/G	0.3	VFA	−0.0002 ± 0.0068	0.982	−0.0093 ± 0.0051	0.0702
				SFA	0.0111 ± 0.0047	**0.0192**	0.0042 ± 0.0033	0.2048
				VFA/SFA	−0.0114 ± 0.0053	**0.0325**	−0.0136 ± 0.0052	**0.0091**
rs6548238	*TMEM18*	T/C	0.09	VFA	−0.0169 ± 0.0111	0.1295	0.0044 ± 0.0085	0.603
				SFA	−0.0174 ± 0.0078	**0.0252**	−0.0010 ± 0.0054	0.8566
				VFA/SFA	0.0004 ± 0.0088	0.9628	0.0052 ± 0.0086	0.5435
rs713586	*RBJ*	C/T	0.46	VFA	0.0089 ± 0.0062	0.1552	0.0055 ± 0.0047	0.2442
				SFA	0.0104 ± 0.0044	**0.0168**	0.0078 ± 0.0030	**0.01**
				VFA/SFA	−0.0016 ± 0.0049	0.7375	−0.0024 ± 0.0048	0.618
rs10195252	*GRB14-COBLL1*	C/T	0.1	VFA	0.0079 ± 0.0104	0.447	−0.0052 ± 0.0079	0.5051
				SFA	0.0162 ± 0.0072	**0.025**	0.0063 ± 0.0051	0.2169
				VFA/SFA	−0.0083 ± 0.0082	0.3079	−0.0115 ± 0.0080	0.1533
rs2535633	*ITIH4-AS1*	C/G	0.41	VFA	0.0099 ± 0.0064	0.1215	−0.0012 ± 0.0049	0.8127
				SFA	0.0012 ± 0.0045	0.7828	−0.0071 ± 0.0031	**0.0225**
				VFA/SFA	0.0087 ± 0.0050	0.0844	0.0060 ± 0.0050	0.225
rs7647305	*ETV5*	T/C	0.06	VFA	−0.0280 ± 0.0136	**0.0391**	−0.0122 ± 0.0103	0.2354
				SFA	−0.0152 ± 0.0095	0.1085	−0.0030 ± 0.0066	0.6501
				VFA/SFA	−0.0127 ± 0.0107	0.2365	−0.0091 ± 0.0105	0.3849
rs2112347	*POC5*	T/G	0.41	VFA	−0.0079 ± 0.0065	0.2239	−0.0111 ± 0.0049	**0.024**
				SFA	−0.0025 ± 0.0046	0.5787	−0.0050 ± 0.0032	0.1133
				VFA/SFA	−0.0055 ± 0.0051	0.2834	−0.0062 ± 0.0050	0.2181
rs9356744	*CDKAL1*	C/T	0.4	VFA	−0.0055 ± 0.0065	0.3962	−0.0106 ± 0.0049	**0.0318**
				SFA	0.0012 ± 0.0045	0.7863	−0.0025 ± 0.0032	0.4284
				VFA/SFA	−0.0068 ± 0.0051	0.1833	−0.0081 ± 0.0050	0.1057
rs206936	*NUDT3*	A/G	0.48	VFA	−0.0076 ± 0.0063	0.2254	−0.0005 ± 0.0048	0.9179
				SFA	−0.0121 ± 0.0044	**0.0061**	−0.0066 ± 0.0031	**0.0318**
				VFA/SFA	0.0045 ± 0.0050	0.3662	0.0061 ± 0.0049	0.2071
rs671	*ALDH2*	A/G	0.22	VFA	−0.036 ± 0.0075	**1.94 × 10**^**−6**^^a^	−0.0224 ± 0.0057	**9.64 × 10**^**−5**^^c^
				SFA	−0.0131 ± 0.0053	**0.0132**	−0.0025 ± 0.0037	0.4966
				VFA/SFA	−0.0229 ± 0.0059	**1.19 × 10**^**−4**^^b^	−0.0199 ± 0.0058	**6.54 × 10**^**−4**^^d^
rs9939609	*FTO*	A/T	0.12	VFA	0.0200 ± 0.0098	**0.0407**	0.0057 ± 0.0074	0.4452
				SFA	0.0131 ± 0.0068	0.0568	0.0021 ± 0.0048	0.6605
				VFA/SFA	0.0072 ± 0.0077	0.3546	0.0037 ± 0.0076	0.6225
rs17782313	*MC4R*	C/T	0.22	VFA	0.0179 ± 0.0076	**0.0178**	0.0010 ± 0.0058	0.8662
				SFA	0.0116 ± 0.0053	**0.0284**	−0.0013 ± 0.0037	0.7179
				VFA/SFA	0.0065 ± 0.0060	0.2742	0.0025 ± 0.005874	0.6686
rs29941	*KCTD15*	C/T	0.24	VFA	0.0069 ± 0.0073	0.3477	−0.0047 ± 0.0056	0.4012
				SFA	0.0134 ± 0.0051	**0.0089**	0.0046 ± 0.0036	0.1945
				VFA/SFA	−0.0067 ± 0.0058	0.2481	−0.0095 ± 0.0057	0.095
rs4823006	*ZNRF3*	A/G	0.46	VFA	−0.0086 ± 0.0064	0.1748	−0.0007 ± 0.0048	0.8879
				SFA	−0.0094 ± 0.0044	**0.0339**	−0.0031 ± 0.0363	0.3114
				VFA/SFA	0.0007 ± 0.0050	0.8899	0.0023 ± 0.0049	0.6346

SNP, single nucleotide polymorphism; Alleles, minor/major alleles; MAF minor allele frequency;SE, standard error; VFA, visceral fat area; SFA, subcutaneous fat area; VFA-SFA, the ratio of visceral fat to subcutaneous fat.

Only SNPs that showed nominal significant associations with traits are shown in Table 3.

*P* values < 0.05 are shown in bold.

Traits were adjusted for age and sex in the additive genetic model 1 and adjusted for age, sex, and BMI in model 2.

^a^Empirical *P* = 0.0003.

^b^Empirical *P* = 0.0057.

^c^Empirical *P* = 0.0043.

^d^Empirical *P* = 0.0345; Empirical *P* values were based on 10000 permutations within each trait.

**Table 4:** Gender differences in how the variants influence fat distribution.

SNP	Gene	Alleles	MAF	Traits	Males		Females		*P* for interaction
					BETA ± SE	*P*	BETA ± SE	*P*	
rs4846567	*LYPLAL1*	T/G	0.3	VFA	−0.0087 ± 0.0083	0.2964	−0.0100 ± 0.0062	0.1063	0.6223
				SFA	−0.0045 ± 0.0045	0.3261	0.0113 ± 0.0046	**0.0144**	**0.0406**
				VFA/SFA	−0.0043 ± 0.0079	0.5866	−0.0216 ± 0.0069	**0.0018**	0.0732
rs713586	*RBJ*	C/T	0.46	VFA	0.0068 ± 0.0075	0.3667	0.0064 ± 0.0058	0.2727	0.9455
				SFA	0.0044 ± 0.0041	0.2868	0.0119 ± 0.0044	**0.0064**	0.2437
				VFA/SFA	0.0081 ± 0.0066	0.2197	0.0039 ± 0.0074	0.5954	0.4853
rs4684854	*PPARG*	G/C	0.08	VFA	−0.0040 ± 0.0143	0.7818	0.0246 ± 0.0111	**0.0262**	0.0812
				SFA	0.0002 ± 0.0078	0.9789	−0.0062 ± 0.0083	0.4533	0.6725
				VFA/SFA	−0.0043 ± 0.0137	0.752	0.0314 ± 0.0124	**0.0111**	**0.0437**
rs2535633	*ITIH4-AS1*	C/G	0.41	VFA	0.0002 ± 0.0078	0.9778	−0.0048 ± 0.0059	0.4173	0.2538
				SFA	−0.0042 ± 0.0043	0.3218	−0.0108 ± 0.0044	**0.0147**	0.1128
				VFA/SFA	0.0043 ± 0.0075	0.5608	0.0061 ± 0.0066	0.3535	0.9261
rs10938397	*GNPDA2*	G/A	0.3	VFA	0.0019 ± 0.0085	0.8279	−0.0028 ± 0.0063	0.6514	0.6623
				SFA	0.0082 ± 0.0046	0.0781	−0.0095 ± 0.0047	**0.0433**	**0.0087**
				VFA/SFA	−0.0065 ± 0.0081	0.4274	0.0066 ± 0.0070	0.3524	0.22
rs4712652	*CASC15 /PRL*	G/A	0.14	VFA	0.0059 ± 0.0113	0.5975	−0.0180 ± 0.0085	**0.0353**	0.1367
				SFA	−0.0006 ± 0.0061	0.9189	0.0001 ± 0.0064	0.9892	0.8117
				VFA/SFA	0.0066 ± 0.0107	0.5361	−0.0176 ± 0.0095	0.0658	0.114
rs206936	*NUDT3*	A/G	0.48	VFA	−0.0058 ± 0.0076	0.4388	0.0016 ± 0.0059	0.7848	0.385
				SFA	−0.0102 ± 0.0041	**0.0132**	−0.0048 ± 0.0044	0.2826	0.2953
				VFA/SFA	0.0044 ± 0.0072	0.5415	0.0064 ± 0.0066	0.3334	0.8493
rs6905288	*VEGFA*	G/A	0.26	VFA	0.0005 ± 0.0091	0.96	−0.0135 ± 0.0066	**0.0395**	0.1641
				SFA	0.0043 ± 0.004956	0.3907	−0.0054 ± 0.0049	0.2733	0.1093
				VFA/SFA	−0.0039 ± 0.0087	0.6499	−0.0079 ± 0.0073	0.2838	0.7499
rs1055144	*NFE2L3*	A/G	0.44	VFA	0.0056 ± 0.0075	0.451	0.0002 ± 0.0059	0.9758	0.5147
				SFA	−0.0080 ± 0.0041	**0.0492**	−0.0005 ± 0.0044	0.9014	0.2794
				VFA/SFA	0.0136 ± 0.0071	0.0559	0.0008 ± 0.0065	0.8997	0.191
rs2075064	*LHX2*	A/G	0.43	VFA	−0.0005 ± 0.0076	0.9428	−0.0040 ± 0.0059	0.4964	0.8214
				SFA	−0.0086 ± 0.0041	**0.0373**	0.0025 ± 0.0044	0.569	**0.0474**
				VFA/SFA	0.0080 ± 0.0072	0.2702	−0.0066 ± 0.0066	0.3183	0.1418
rs4074134	*BDNF*	A/G	0.44	VFA	−0.0024 ± 0.0077	0.7533	0.0118 ± 0.0059	**0.0462**	0.0951
				SFA	−0.0020 ± 0.0042	0.6253	0.0074 ± 0.0044	0.0979	0.0703
				VFA/SFA	−0.0004 ± 0.0073	0.9601	0.0044 ± 0.0066	0.5033	0.6242
rs3817334	*MTCH2*	T/C	0.33	VFA	−0.0075 ± 0.0083	0.3651	0.0044 ± 0.0063	0.4844	0.3136
				SFA	−0.0091 ± 0.0045	**0.0427**	−0.0039 ± 0.0047	0.4057	0.506
				VFA/SFA	0.0015 ± 0.0079	0.8475	0.0081 ± 0.0070	0.2505	0.5769
rs671	*ALDH2*	A/G	0.22	VFA	−0.0529 ± 0.0093	**1.75 × 10**^**−8**^^a^	0.0025 ± 0.0069	0.7173	**9.88 × 10**^**−8**^
				SFA	−0.0040 ± 0.0051	0.4345	−0.0003 ± 0.0051	0.9544	0.3102
				VFA/SFA	−0.049 ± 0.0089	**4.43 × 10**^**−8**^^a^	0.0027 ± 0.0077	0.7241	**4.67 × 10**^**−6**^
rs9568856	*OLFM4*	A/G	0.31	VFA	0.0003 ± 0.0083	0.9693	0.0011 ± 0.0065	0.8642	0.9478
				SFA	0.0025 ± 0.0045	0.5873	0.0096 ± 0.0048	**0.0468**	0.3671
				VFA/SFA	−0.0022 ± 0.0079	0.7839	−0.0086 ± 0.0072	0.2326	0.5215
rs10146997	*NRXN3*	G/A	0.003	VFA	−0.0886 ± 0.0879	0.3135	0.0908 ± 0.0497	0.0678	**0.0303**
				SFA	−0.0012 ± 0.0479	0.9797	0.0739 ± 0.0372	**0.047**	0.1384
				VFA/SFA	−0.0853 ± 0.0838	0.3089	0.0169 ± 0.0556	0.7606	0.2434
rs1424233	*MAF*	G/A	0.32	VFA	−0.0084 ± 0.0081	0.2981	0.0110 ± 0.0062	0.0785	0.0582
				SFA	−0.0015 ± 0.0044	0.7351	−0.0032 ± 0.0047	0.4908	0.7703
				VFA/SFA	−0.0068 ± 0.0077	0.3792	0.0140 ± 0.0070	**0.0443**	**0.0443**

SNP, single nucleotide polymorphism; Alleles, minor/major alleles; MAF minor allele frequency; SE, standard error; VFA, visceral fat area; SFA, subcutaneous fat area; VFA-SFA, the ratio of visceral fat to subcutaneous fat.

Only SNPs that showed nominal significant associations with traits are shown in Table 4.

*P* values <0.05 are shown in bold.

Traits were adjusted for age and BMI in the additive genetic model.

^a^Empirical *P* = 1 × 10-4; Empirical *P* values were based on 10000 permutations within each trait.
