# Supplementary material for: Effects of Obesity Related Genetic Variations on Visceral and Subcutaneous Fat Distribution in a Chinese Population
Source: Sci Rep. 2016 Feb 5;6:20691. doi: 10.1038/srep20691 (PMC4742921; doi:10.1038/srep20691)

Effects of Obesity Related Genetic Variations on Visceral and Subcutaneous Fat Distribution in a Chinese Population

Tao Wang[1#], Xiaojing Ma[1#], Danfeng Peng[1], Rong Zhang[1], Xue Sun[1], Miao Chen[1], Jing Yan[1], Shiyun Wang[1], Dandan Yan[1], Zhen He[1], Feng Jiang[1], Yuqian Bao[1], Cheng Hu[1,2]* & Weiping Jia[1]*

1. Shanghai Diabetes Institute, Shanghai Key Laboratory of Diabetes Mellitus, Shanghai Clinical Center for Diabetes, Shanghai Jiao Tong University Affiliated Sixth People's Hospital, Shanghai, 200233, China

2. Institute for Metabolic Diseases, Shanghai Jiao Tong University Affiliated Sixth People's Hospital South Campus, Shanghai, 201406, China

Tao Wang and Xiaojing Ma contributed equally to this article.

Corresponding authors: Cheng Hu and Weiping Jia

Shanghai Diabetes Institute, Shanghai Jiao Tong University Affiliated Sixth People's Hospital, 600 Yishan Road, Shanghai, 200233, China

Emails: Cheng Hu (alfredhc@sjtu.edu.cn)

Weiping Jia (wpjia@sjtu.edu.cn)

Tel: +86 21 24058924

Fax:86-21-64368031

Supplemental Tables

Supplemental Table 1 Characteristics of 57 established SNPs

SNP	Gene	Chr	Position	Minor/ Major allele	MAF	Traits	First Author	Reference	Risk allele	Reported beta	Power (%)
rs2568958	*NEGR1*	1	72299433	G/A	0.08	BMI	Thorleifsson	8	A	0.03	5
rs1514175	*TNNI3K*	1	74525960	C/T	0.23	BMI	Speliotes	27	A	0.07	8
rs1555543	*PTBP2*	1	96479241	A/C	0.13	BMI	Speliotes	27	C	0.06	6
rs984222	*TBX15-WARS2*	1	118961220	C/G	0.41	WHR	Heid	10	G	0.031	50
rs1011731	*PIGC-DNM3*	1	172377408	C/T	0.12	WHR	Heid	10	G	0.026	19
rs574367	*SEC16B*	1	177904075	T/G	0.2	BMI	Thorleifsson	8	T	0.06	8
rs4846567	*LYPLAL1*	1	219577375	T/G	0.3	WHR	Heid	10	G	0.032	45
rs11118316	*LYPLAL1*	1	217723786	-	-	VFA/SFA	Fox	12	A	NR	-
rs6548238	*TMEM18*	2	634905	T/C	0.09	BMI	Willer	9	C	0.26	25
rs1057001	*TRIB2*	2	12741652	T/A	0.15	VFA	Nakayama	31	T	0.1	5
rs713586	*RBJ*	2	24935139	C/T	0.46	BMI	Speliotes	27	C	0.14	36
rs887912	*FANCL*	2	59075742	A/G	0.002	BMI	Speliotes	27	T	0.1	6
rs1659258	*THNSL2*	2	88360069	G/A	0.1	VFA	Fox	12	A	NR	-
rs10195252	*GRB14-COBLL1*	2	164656581	C/T	0.1	WHR	Heid	10	T	0.036	28
rs2943650	*IRS1*	2	226241205	C/T	0.07	body fat percentage	Kilpelainen	11	T	0.16	7
rs4684854	*PPARG*	3	12447383	G/C	0.08	WHR	Randal	23	C	0.037	25
rs2535633	*ITIH4-AS1*	3	52825614	C/G	0.41	BMI	Wen	21	G	0.03	44
rs7647305	*ETV5*	3	186116501	T/C	0.06	BMI	Thorleifsson	8	C	0.04	5
rs10938397	*GNPDA2*	4	45180510	G/A	0.3	BMI	Willer	9	G	0.18	47
rs11743303	*MAP3K1*	5	56564125	G/A	0.11	WC	Randal	23	G	0.031	12

rs2112347	*POC5*	5	75719417	T/G	0.41	BMI	Speliotes	27	T	0.1	21
rs10478424	*HSD17B4*	5	119453325	A/T	0.44	WHR	Randal	23	A	0.039	74
rs6861681	*CPEB4*	5	173935455	A/G	0.09	WHR	Heid	10	A	0.019	11
rs6931262	*RREB1*	6	7217284	T/C	0.2	WHR	Liu	24	T	0.06	89
rs9356744	*CDKAL1*	6	20685255	C/T	0.4	BMI	Wen	20	T	0.03	43
rs4712652	*CASC15 /PRL*	6	22078386	G/A	0.14	extreme obesity	Meyre	14	A	0.83	-
rs206936	*NUDT3*	6	34335092	A/G	0.48	BMI	Speliotes	27	G	0.06	9
rs6905288	*VEGFA*	6	43791136	G/A	0.26	WHR	Heid	10	A	0.039	60
rs987237	*TFAP2B*	6	50835337	G/A	0.17	BMI	Speliotes	27	G	0.11	12
rs1055144	*NFE2L3*	7	25831489	A/G	0.44	WHR	Heid	10	T	0.043	78
rs10261878	*MIR148A*	7	25910925	A/C	0.03	BMI	Monda	22	C	0.032	5
rs545854	*MSRA*	8	10002570	C/G	0.43	WC	Lindgren	7	G	0.43	41
rs10968576	*LRRN6C-LINGO2*	9	28414341	G/A	0.21	BMI	Speliotes	27	G	0.11	12
rs11142387	*KLF9*	9	70383416	C/A	0.32	BMI	Okada	26	C	0.03	6
rs2075064	*LHX2*	9	124021568	A/G	0.43	WC	Liu	24	C	0.07	6
rs11191580	*NT5C2*	10	103146454	C/T	0.29	BMI	Wen	21	C	0.03	38
rs4929949	*RPL27A*	11	8583046	T/C	0.41	BMI	Speliotes	27	C	0.06	10
rs4074134	*BDNF*	11	27625738	A/G	0.44	BMI	Thorleifsson	8	G	0.04	7
rs3817334	*MTCH2*	11	47629441	T/C	0.33	BMI	Speliotes	27	T	0.06	10
rs7138803	*FAIM2*	12	49853685	A/G	0.28	BMI	Thorleifsson	8	A	0.12	12
rs1443512	*HOXC13*	12	53948900	A/C	0.2	WHR	Heid	10	A	0.03	36
rs671	*ALDH2*	12	111803962	A/G	0.22	BMI	Wen	21	G	0.038	49
rs2074356	*ALDH2-HECTD4*	12	112207597	T/C	0.15	WHR	Cho	17	C	0.005	63
rs4771122	*MTIF3*	13	27446043	G/A	0.18	BMI	Speliotes	27	G	0.09	13
rs9568856	*OLFM4*	13	53490846	A/G	0.31	extreme obesity	Bradfield	15	A	1.21	-
rs534870	*SPRY2*	13	80385072	G/A	0.42	body fat	Kilpelainen	11	A	0.14	10

						percentage					
rs10146997	*NRXN3*	14	79478819	G/A	0.003	WC	Heard-Costa	6	G	0.59	38
rs12597579	*GP2*	16	20246545	T/C	0.27	BMI	Wen	20	C	0.04	58
rs4788102	*SH2B1*	16	28862077	A/G	0.13	BMI	Thorleifsson	8	A	0.15	14
rs9939609	*FTO*	16	53786615	A/T	0.12	BMI	Frayling	4	A	0.33	47
rs1424233	*MAF*	16	79648854	G/A	0.32	extreme obesity	Meyre	14	A	0.72	-
rs1805081	*NPC1*	18	23560468	G/A	0.24	extreme obesity	Meyre	14	A	1.41	-
rs17782313	*MC4R*	18	60183864	C/T	0.22	BMI	Loos	5	C	0.2	33
rs29941	*KCTD15*	19	33818627	C/T	0.24	BMI	Thorleifsson	8	C	0.06	9
rs2287019	*QPCTL*	19	45698914	T/C	0.18	BMI	Speliotes	27	C	0.15	17
rs3810291	*TMEM160*	19	47065746	A/G	0.29	BMI	Speliotes	27	A	0.09	16
rs4823006	*ZNRF3*	22	29055683	A/G	0.46	WHR	Heid	10	A	0.019	23

SNP, single nucleotide polymorphism; Chr, chromosome; MAF, minor allele frequency in our sample of Chinese; WC, waist circumference; WHR, waist to hip ratio; VFA/SFA, the ratio of visceral fat to subcutaneous fat. NR, not reported.

The allele that increased level of traits is denoted as risk allele.

Supplemental Table 2 Gender differences in how the variants influence fat distribution irrespective of BMI

SNP	Gene	Alleles	MAF	Traits	Males		Females		*P* for interaction
					BETA ±SE	*P*	BETA ±SE	*P*	
rs1011731	*PIGC-DNM3*	C/T	0.12	VFA	0.0321 ±0.0155	**0.0392**	-0.0079 ±0.0119	0.5059	**0.0294**
				SFA	0.0173 ±0.0101	0.0886	-0.0022 ±0.0090	0.805	0.1316
				VFA/SFA	0.0147 ±0.0113	0.1949	-0.0059 ±0.0103	0.5666	0.1531
rs574367	*SEC16B*	T/G	0.2	VFA	0.0217 ±0.0134	0.1042	0.0227 ±0.0095	**0.0168**	0.9764
				SFA	0.0207 ±0.0087	**0.0173**	0.0151 ±0.0072	**0.0363**	0.6029
				VFA/SFA	0.0009 ±0.0097	0.923	0.0076 ±0.0082	0.3559	0.6178
rs4846567	*LYPLAL1*	T/G	0.3	VFA	-0.0038 ±0.0112	0.7363	0.0011 ±0.0081	0.8939	0.8266
				SFA	-0.0007 ±0.0073	0.9261	0.0197 ±0.0062	**0.0015**[b]	**0.0398**
				VFA/SFA	-0.0032 ±0.0081	0.6974	-0.0188 ±0.0070	**0.0073**	0.1185
rs6548238	*TMEM18*	T/C	0.09	VFA	-0.0024 ±0.0182	0.897	-0.0279 ±0.0135	**0.0392**	0.2941
				SFA	-0.0032 ±0.0119	0.7843	-0.0287 ±0.0103	**0.0052**	0.1159
				VFA/SFA	0.0008 ±0.0132	0.9511	0.0006 ±0.0117	0.9577	0.9545
rs713586	*RBJ*	C/T	0.46	VFA	0.0062 ±0.0101	0.5368	0.0124 ±0.0076	0.1052	0.5829
				SFA	0.0039 ±0.0066	0.5496	0.0166 ±0.0058	**0.0044**	0.1395
				VFA/SFA	0.0235 ±0.0106	**0.0267**	0.0103 ±0.0099	0.2997	0.3676
rs4684854	*PPARG*	G/C	0.08	VFA	0.0020 ±0.0192	0.9152	0.0100 ±0.0145	0.4936	0.6748
				SFA	0.0048 ±0.0125	0.7011	-0.0177 ±0.0110	0.1091	0.1945
				VFA/SFA	-0.0029 ±0.0140	0.8353	0.0283 ±0.0125	**0.0242**	0.0847
rs7647305	*ETV5*	T/C	0.06	VFA	-0.0165 ±0.0223	0.4601	-0.0341 ±0.0164	**0.0378**	0.5504
				SFA	-0.0135 ±0.0145	0.352	-0.0153 ±0.0125	0.2205	0.9492
				VFA/SFA	-0.0031 ±0.0162	0.8484	-0.0185 ±0.0142	0.1908	0.4961
rs6861681	*CPEB4*	A/G	0.09	VFA	0.0254 ±0.0181	0.1599	-0.0264 ±0.0134	0.0502	**0.0335**
				SFA	0.0080 ±0.0118	0.4953	0.0002 ±0.0102	0.9808	0.7015

				VFA/SFA	0.0172±0.0132	0.191	-0.0263±0.0116	**0.0236**	**0.0199**
rs206936	*NUDT3*	A/G	0.48	VFA	-0.0092±0.0101	0.3664	-0.0083±0.0078	0.288	0.9747
				SFA	-0.0127±0.0066	0.0542	-0.0124±0.0059	**0.0358**	0.9787
				VFA/SFA	0.0036±0.0073	0.6235	0.0042±0.0067	0.5351	0.9854
rs987237	*TFAP2B*	G/A	0.17	VFA	0.0278±0.0133	**0.0368**	0.0004±0.0101	0.9676	0.1012
				SFA	0.0128±0.0087	0.141	0.0070±0.0077	0.3639	0.6226
				VFA/SFA	0.0152±0.0097	0.1175	-0.0070±0.0087	0.4226	0.0917
rs1055144	*NFE2L3*	A/G	0.44	VFA	0.01084±0.0100	0.2789	0.0051±0.0077	0.5094	0.6996
				SFA	-0.0040±0.0065	0.5402	0.0036±0.0058	0.5354	0.3596
				VFA/SFA	0.0148±0.0073	**0.0418**	0.0016±0.0066	0.8146	0.1972
rs10261878	*MIR148A*	A/C	0.03	VFA	-0.0274±0.0293	0.3502	0.0112±0.0234	0.6314	0.3838
				SFA	-0.0377±0.0191	**0.049**	0.0008±0.0178	0.9661	0.1702
				VFA/SFA	0.0104±0.0213	0.6273	0.0109±0.0202	0.5913	0.9183
rs2075064	*LHX2*	A/G	0.43	VFA	-0.0089±0.0102	0.3816	-0.0079±0.0077	0.3078	0.9702
				SFA	-0.0150±0.0066	**0.0237**	-0.0005±0.0059	0.9362	0.1057
				VFA/SFA	0.0060±0.0074	0.4168	-0.0075±0.0067	0.2626	0.1661
rs671	*ALDH2*	A/G	0.22	VFA	-0.0748±0.0125	**2.56×10^{-9}**[a]	-0.0057±0.0090	0.5294	**2.54×10^{-6}**
				SFA	-0.0209±0.0082	**0.011**	-0.0069±0.0069	0.3182	0.1574
				VFA/SFA	-0.0539±0.0091	**3.48×10^{-9}**[a]	0.0011±0.0078	0.8872	**2.54×10^{-6}**
rs534870	*SPRY2*	G/A	0.42	VFA	0.0034±0.0104	0.7417	0.0032±0.0078	0.6822	0.8826
				SFA	-0.0080±0.0068	0.2391	0.0121±0.0059	**0.0396**	**0.0191**
				VFA/SFA	0.0115±0.0076	0.1302	-0.0090±0.0067	0.1782	0.0574
rs17782313	*MC4R*	C/T	0.22	VFA	0.0005±0.0120	0.9653	0.0341±0.0094	**2.93×10^{-4}**[c]	**0.0106**
				SFA	0.0063±0.0078	0.422	0.0166±0.0072	**0.0207**	0.241
				VFA/SFA	-0.0057±0.0087	0.5112	0.0179±0.0081	**0.0279**	**0.026**
rs4823006	*ZNRF3*	A/G	0.46	VFA	-0.0154±0.0103	0.1369	-0.0020±0.0077	0.7962	0.2924

SFA	-0.0145 ±0.0067	**0.0317**	-0.0048 ±0.0059	0.4134	0.2783
VFA/SFA	-0.0009 ±0.0075	0.9029	0.0026 ±0.0067	0.6963	0.7244

SNP, single nucleotide polymorphism; Alleles, minor/major alleles; MAF, minor allele frequency; SE, standard error; VFA, visceral fat area; SFA, subcutaneous fat area; VFA/SFA, the ratio of visceral fat to subcutaneous fat.

Only SNPs that showed nominal significant associations with traits are shown in Supplemental Table 2.

P values<0.05 are shown in bold.

Traits were adjusted for age in the additive genetic model.

[a] Empirical *P*=1×10-4, [b] Empirical *P*= 0.0778, [c] Empirical *P*=0.0166; Empirical *P* values were based on 10000 permutations within each trait.

Supplementary Figure legend

Supplementary Figure 1.Linkage disequilibrium map for each of the regions in the Chinese populations. Shades of red demonstrate the strength of the pairwise linkage disequilibrium based on r^2 and number shown is r^2 of each SNP pair expressed as a percentage. The SNPs tested in our study are marked with red blox. rs887912 in *FANCL* is not included for minor alelle frequency (MAF=0 in Hapmap data) in Chinese populations. rs11118316 in *LYPALl*, rs4712652 in *CASC15* and rs4929949 in *RPL27A* are substuted with another SNPs (r^2>0.84) in same region. The SNPs in the same region (e.g., rs671 and rs2074356 in *ALDH2* and rs4846567 and rs4623705 in *LYPLA1*) are shown in one map.

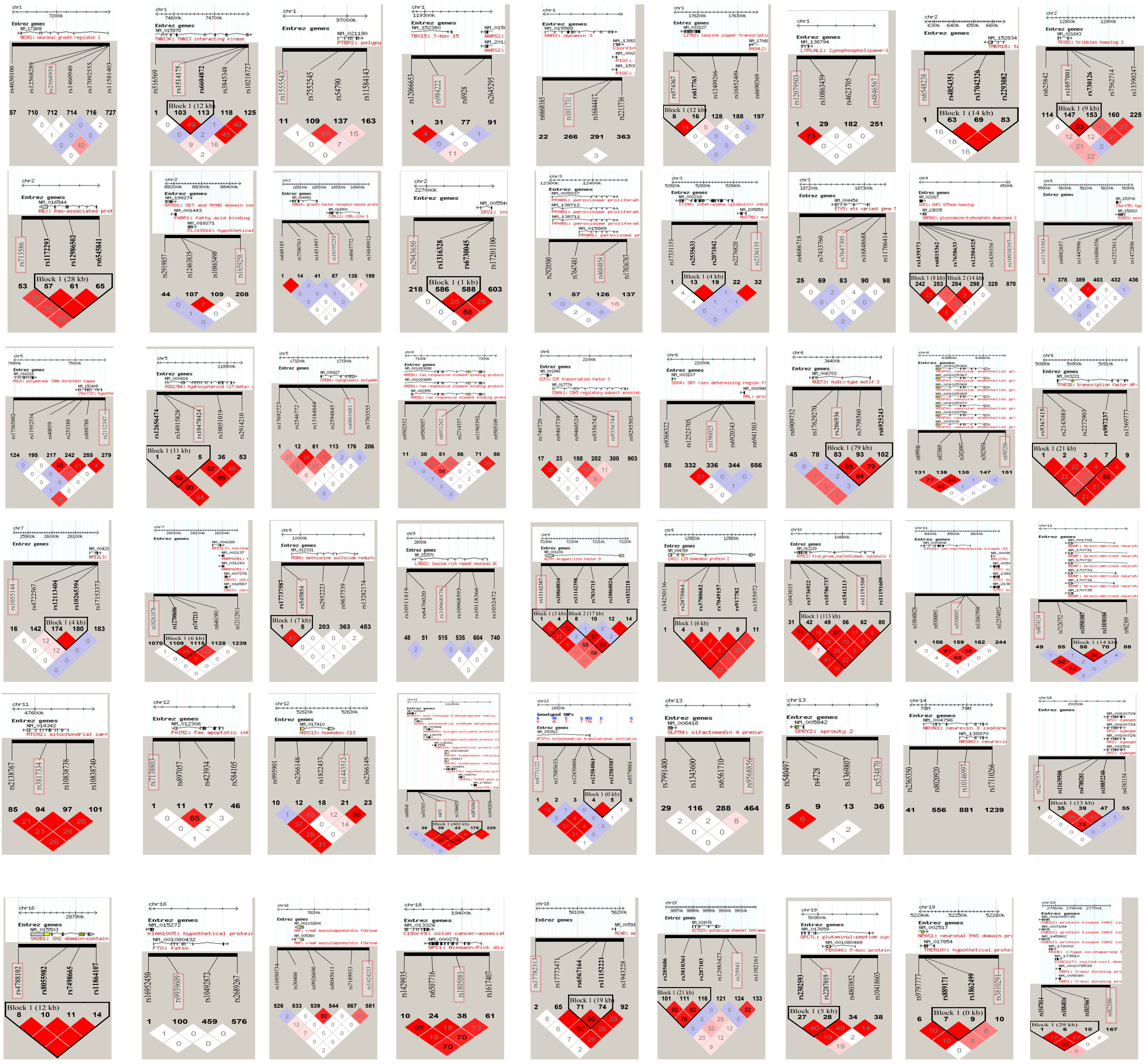

Supplement: Supplementary Information [file srep20691-s1.pdf]
